# Antibiotic treatment duration for culture-negative sepsis in the pediatric intensive care unit

**DOI:** 10.1017/ash.2023.502

**Published:** 2023-12-22

**Authors:** Kelsey Wehrenberg, Michelle Mitchell, Tracy Zembles, Ke Yan, Liyun Zhang, Nathan Thompson

**Affiliations:** 1 Section of Critical Care, Department of Pediatrics, University of Florida, Gainesville, FL, USA; 2 Section of Critical Care, Department of Pediatrics, Medical College of Wisconsin, Milwaukee, WI, USA; 3 Section of Infectious Diseases, Department of Pediatrics, Medical College of Wisconsin, Milwaukee, WI, USA; 4 Department of Enterprise Safety, Children’s Wisconsin, Milwaukee, WI, USA; 5 Section of Quantitative Health Sciences, Department of Pediatrics, Medical College of Wisconsin, Milwaukee, WI, USA

## Abstract

**Objective:**

Sepsis remains a leading cause of morbidity and mortality in children. There is very limited guidance for sepsis treatment when cultures remain negative. This study sought to determine the effectiveness of short versus long course of antibiotics when treating culture-negative sepsis and assess for subsequent multidrug-resistant organism (MDRO) acquisition.

**Design:**

Retrospective cohort study.

**Setting:**

Quaternary academic children’s hospital.

**Patients:**

Pediatric intensive care unit (ICU) patients with culture-negative sepsis receiving a minimum of 72 hours of antibiotics.

**Methods:**

Patients found to have culture-negative sepsis from January 2017 to May 2020 were divided into two groups: short and long course of antibiotics. Various demographic and laboratory results were collected for each subject as available. Primary outcomes included mortality and lengths of stay. The secondary outcome was subsequent acquisition of a new MDRO.

**Results:**

Eighty-six patients were treated for culture-negative sepsis with 43 patients in both the short- (< or = 7 days) and long-course (>7 days) treatment cohorts. Patients who received a short course of antibiotics had a lower overall mortality than those who received a long course (9.3% vs 25.6% *p* = 0.047), but there was no difference in 30-day mortality (*p* > 0.99). Patients in the short-course group had a shorter hospital length of stay (22 vs 30 days *p* = 0.018). New MDROs were found in 10% of all patients.

**Conclusions:**

Treatment of culture-negative sepsis with short-course antibiotics was not associated with worse outcomes in ICU patients. These findings warrant further investigation with a larger, prospective, multi-center study.

## Introduction

Sepsis is a life-threatening condition requiring prompt treatment with initiation of broad-spectrum antibiotics.^1^ Antibiotics should then be tailored to a specific organism if one is identified from cultures.^2^ However, when cultures remain negative and there is no clear focus of infection, there is no organism to target with narrowed antimicrobial coverage. This often leaves providers with high suspicion for infection the difficult decision regarding choice of antibiotic and duration. The choice of overly broad and/or prolonged exposure to broad-spectrum antibiotics in these cases can contribute to the development of multidrug-resistant organisms (MDROs).^3^

The CDC estimates 2.8 million infections in the United States each year are caused by drug-resistant bacteria.^4^ Intensive care unit (ICU) patients are particularly vulnerable to the acquisition of MDROs given severe illness, immunosuppressed states, exposure to broad-spectrum antibiotics, and prolonged hospital stays.^5,6^ Infections with resistant organisms can lead to poor outcomes, including death.

There is currently very limited literature on culture-negative sepsis, especially in the pediatric population. Neonatal literature has addressed decreasing antibiotic durations with the introduction of 48-hour hard stops to rule out sepsis and a shorter duration of antibiotics (5 days) for pneumonia and culture-negative sepsis.^7^ Adult literature concentrates on trends (incidence of culture-negative sepsis) and outcomes (mortality, length of stay, acute organ dysfunction, etc.) of culture-negative versus culture-positive sepsis with conflicting data.^8–11^ Currently, no literature in the pediatric population assesses treatment duration of culture-negative sepsis and if duration of treatment is associated with subsequent development of MDROs.

This study aims to investigate antibiotic treatment duration in culture-negative sepsis in the pediatric population. We hypothesized that shorter courses of antibiotics (< or = 7 days) would not compromise clinical outcomes compared to longer courses of antibiotics in the treatment of culture-negative sepsis. We then further investigated if short versus long courses of antibiotics are associated with a decreased occurrence of MDRO acquisition.

## Methods

Our hospital’s Institutional Review Board approved this retrospective single-center chart review of pediatric patients ages 0–18 years admitted to the pediatric ICU at a quaternary free-standing children’s hospital for treatment of culture-negative sepsis from January 2017 through May 2020. Patients were included if they received a minimum of 72 hours of antibiotics for presumed bacterial infection despite negative cultures and no specific identified focus of infection. Patients on extracorporeal membrane oxygenation (ECMO) who received broadened antibiotic coverage for culture-negative sepsis were included as were patients with positive cultures documented by the clinical team as contaminants or colonization. Patients were excluded if any of the following applied: subsequent cases of culture-negative sepsis (ie only the first case of culture-negative sepsis for an individual patient was included), diagnosis with an infection with general treatment duration guidelines (ie pneumonia including aspiration pneumonia, acute otitis media, cellulitis, toxic shock syndrome, etc.), antibiotic prophylaxis while on ECMO, antibiotics for post-surgical management, acute liver failure, febrile neutropenia (ANC < or = 500), or admission to the neonatal ICU. Febrile neutropenia patients were excluded because they were managed according to evidence-based treatment guidelines developed by the hospital’s oncology service. The initial cohort of patients was identified by either a sepsis best practice advisory (BPA) alert in the electronic medical record or a pharmacy medication database search based on administration of antibiotics in patients with a negative blood culture. The sepsis BPA can fire at any time during admission based on WBC count/abnormal temperature with two other findings including vital signs, physical exam findings, or patients with high-risk conditions. We reviewed each patient’s chart for confirmation of presumed bacterial infection without an identifiable source.

Microbiologic data collected included blood, urine, endotracheal, tracheal, cerebral spinal fluid, and wound cultures as well as viral studies at the time of the sepsis event. Previous MDROs and their minimum inhibitory concentrations (MIC) were also noted. Laboratory data such as WBC, platelet count, lactate, procalcitonin, and CRP at the start of the sepsis event were collected when available. Additionally, clinical data including fever, use of mechanical ventilation, inotropic medication use, and outcome variables including mortality (both within 30 days of the start of the sepsis event and at any point (overall mortality)), PICU length of stay (LOS), hospital LOS, and MDRO acquisition over a 6-month period following the sepsis event were collected. The hospital Virtual Pediatric Systems database was used for obtaining demographic information, Pediatric Risk of Mortality (PRISM) 3, Paediatric Index of Mortality (PIM)-2, and mortality data.^8,9^ PRISM 3 and PIM-2 scores are validated mortality risk estimate tools from large databases using a variety of factors collected at admission based on a variety of age-stratified physiologic and additional factors.^12,13^

Study data were collected and managed using REDCap electronic data capture tools, hosted at our institution. Primary outcomes included mortality, ICU LOS, and hospital LOS to determine if a short antibiotic course is as effective as a longer one. Subjects were divided into two groups based on length of therapy (LOT): those who received shorter duration of antibiotic treatment (less than or equal to 7 days) and those received longer duration of antibiotics (greater than 7 days). In addition, individual antibiotic durations were added together to calculate days of therapy (DOT). The secondary outcome was the acquisition of a new MDRO over a 6-month period following the sepsis event. New MDRO was defined as either a new organism or 4-fold increase in the MIC of prior MDROs.^14^ Summary statistics, median, interquartile range (IQR), frequency, and percentage were used to report data. To compare the differences between two groups, a chi-square or Fisher’s exact test was used for categorical variables, and the Mann-Whitney test was used for continuous variables. Secondary analysis on ICU LOS and hospital LOS was done adjusting for mortality during the hospitalization. In this method, mortality was treated as a competing risk, which precluded the occurrence of the other event of interest, which was discharge from the ICU or hospital. Additionally, multivariable analyses were completed using a competing risk regression for hospital length of stay and a logistic regression for mortality. Both regression models were used to adjust for covariates: mechanical ventilation, pulmonary disease, PRISM 3 scores, and hospital length of stay prior to the sepsis event. A two-sided *p*-value <0.05 was considered statistically significant. SAS 9.4 (SAS Institute ln, Cary, NC), SPSS (version 26), and R version 3.5.3 were used for all the analyses.

## Results

**Figure 1.**
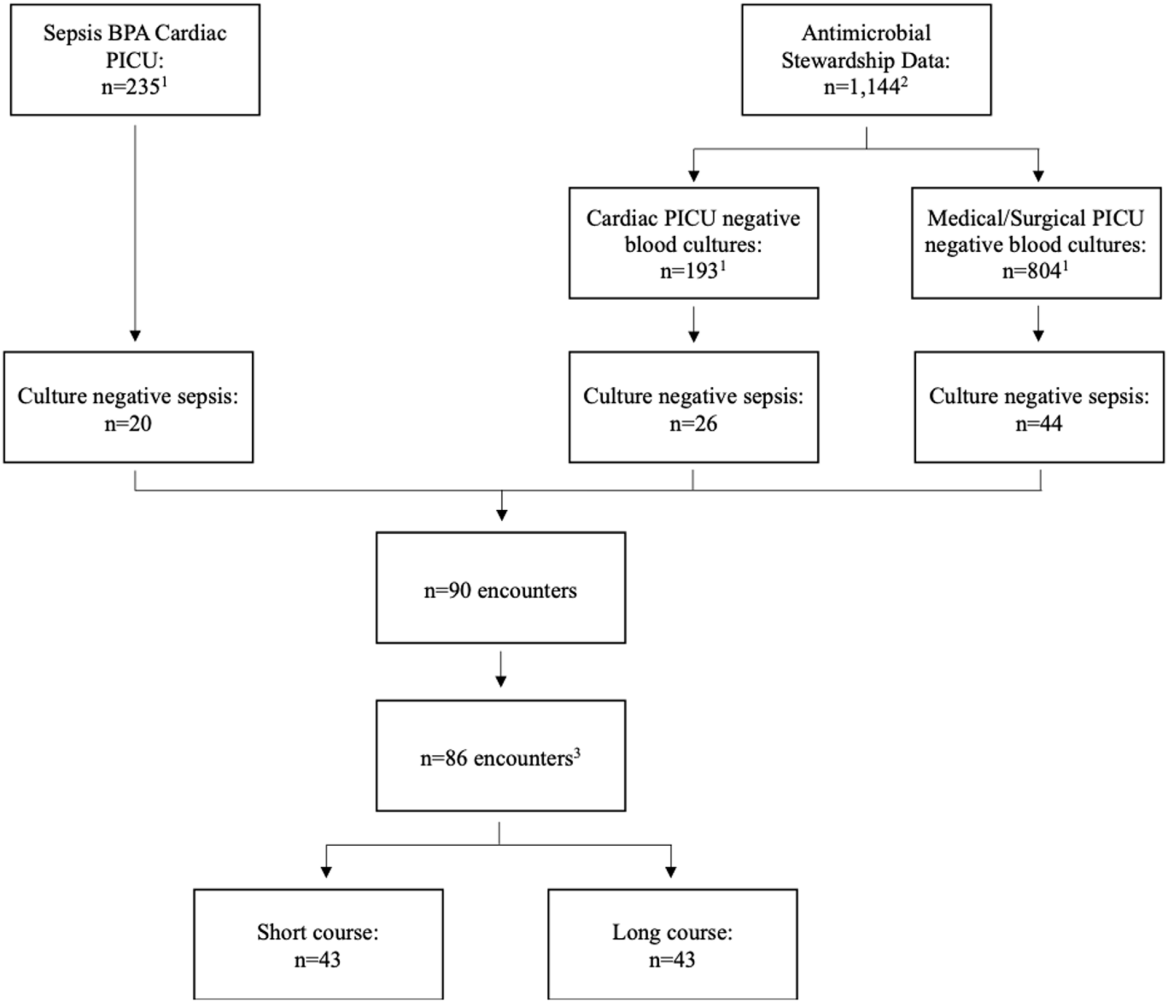
Identification of culture-negative sepsis cohort. 1. Removal of patients with identified/suspected source of infection. 2. Removal of patients found to have a positive blood culture. 3. Removal of subsequent hospitalizations.

We identified 86 patients with culture-negative sepsis during the study period (Figure 1). Forty-three patients were treated with antibiotics for less than or equal to 7 days (short course), and forty-three patients were treated for greater than 7 days (long course). Median (IQR) LOT was 5.65 days (3.28, 6.52) for the short course and 8.54 days (7.71, 9.81) for the long course. Demographics (Table 1) were not significantly different between the two groups. Patients with underlying pulmonary disease were more likely to receive a long course of antibiotics (*p* = 0.040), but other co-morbidities were not significantly different between the two groups (Table 1).

**Table 1.** Characteristics between short and long antibiotic course treatment groups. Significant *p*-value is <0.05

	Less than or equal to 7 days (*n* = 43)	Greater than 7 days (*n* = 43)	*p*-value
Age (median in years (IQR))	1.12 (0.38, 11.76)	0.73 (0.22, 4.9)	0.23
Gender			
Male, *n* (%)	20 (46.51)	20 (46.51)	>0.99
Female, *n* (%)	23 (53.49)	23 (53.49)	
Prematurity, *n* (%)	2 (4.65)	5 (11.63)	0.43
Pulmonary disease, *n* (%)	10 (23.26)	19 (44.19)	**0.040**
Congenital heart disease, *n* (%)	18 (41.86)	26 (60.47)	0.08
Acquired heart disease, *n* (%)	5 (11.63)	3 (6.98)	0.73
Neuromuscular disease, *n* (%)	3 (6.98)	1 (2.33)	0.62
Seizure disorder, *n* (%)	12 (27.91)	7 (16.28)	0.19
Neurological disorder, *n* (%)	17 (39.53)	9 (20.93)	0.10
Hematologic disorder, *n* (%)	4 (9.30)	1 (2.33)	0.36
Congenital abnormalities, *n* (%)	12 (27.91)	19 (44.19)	0.12
Other co-morbidities, *n* (%)	22 (51.16)	16 (37.21)	0.19
PIM-2 score, Median (IQR)	−4.20 (−4.69, −3.14)	−4.22 (−4.69, −3.16)	0.88
PRISM 3 score, Median (IQR)	3 (0, 7)	6 (2, 13)	0.060

PIM-2 and PRISM 3 scores were not statistically different between the short and long course of antibiotics groups, but PRISM 3 scores did trend toward significant. Patients in the longer course group were more likely to have a longer hospital length of stay prior to the sepsis event (*p* = 0.011) and require mechanical ventilation (*p* = 0.031). However, short and long-course groups, however, did not have significant difference in the need for inotropic medications or ECMO (Table 2).

**Table 2.** Clinical features at presentation of patients with culture-negative sepsis

	Less than or equal to 7 days (*n* = 43)	Greater than 7 days (*n* = 43)	*p*-value
Fever (>38.5 °C), *n* (%)	27 (62.79)	23 (53.49)	0.38
Inotropic medications, *n* (% requiring)	31 (72.09)	30 (69.77)	0.81
Mechanical ventilation, *n* (% requiring)	18 (41.86)	28 (65.12)	**0.031**
ECMO, *n* (% requiring)	2 (4.65)	6 (13.95)	0.26
WBC 10^3^/μL, Median (IQR)	12.2 (6.82, 17.70)	14.8 (9.7, 21.4)	0.09
Lactate mg/dL, Median (IQR)^a^	17.00 (9.00, 28.00)	17.00 (11.00, 31.00)	0.66
Procalcitonin ng/dL, Median (IQR)^a^	1.05 (0.24, 12.64)	5.51 (0.27, 16.81)	0.35
CRP mg/dL, Median (IQR)^a^	4.3 (1.5, 16.5)	3.8 (1.6, 7.6)	0.64
Hospital LOS prior to sepsis event, Median days (IQR)	14.08 (6.43, 31.94)	23.68 (12.26, 75.59)	**0.011**

Significant *p*-value is <0.05.

a There are some missing data.

Analysis of antibiotic usage revealed the five most frequently used antibiotics to be vancomycin, cefepime, piperacillin/tazobactam, ceftriaxone, and clindamycin. There was no statistical difference between short and long-course groups in regard to specific antibiotics used (Table 3).

**Table 3.** Most frequent antibiotics used for patients with culture-negative sepsis

Antibiotic, *n* (%)	Overall (*n* = 86)	Less than or equal to 7 days (*n* = 43)	Greater than 7 days (*n* = 43)	*p*-value
Vancomycin	63 (73.26)	32 (74.42)	31 (72.09)	0.81
Cefepime	53 (61.63)	26 (60.47)	27 (62.79)	0.83
Piperacillin/tazobactam	26 (30.23)	12 (27.91)	14 (32.56)	0.82
Ceftriaxone	23 (26.74)	13 (30.23)	10 (23.26)	0.47
Clindamycin	14 (16.28)	6 (13.95)	8 (18.6)	0.56

Significant *p*-value is <0.05.

Overall mortality across both groups was 15 (17%). Patients who received a short course of antibiotics had a lower mortality at any time compared to those in the long-course group (9.3% vs 25.6% *p* = 0.047). This held true in a multivariable regression analysis using mechanical ventilation, pulmonary disease, PRISM 3, and prior hospital LOS as covariates (*p* = 0.035, OR = 4.37, 95% CI [1.11, 17.23]). However, all-cause 30-day mortality from the start of the sepsis event was not significantly different between the two groups (7.0% vs 7.0% *p* > 0.99) (Table 4).

**Table 4.** Outcome measures for culture-negative sepsis. Significant *p*-value is <0.05

	Less than or equal to 7 days (*n* = 43)	Greater than 7 days (*n* = 43)	*p*-value
Overall mortality (17%), *n* (%)	4 (9.30)	11 (25.58)	**0.047**
30-day mortality, *n* (%)	3 (6.98)	3 (6.98)	>0.99
Overall PICU length of stay, median days (IQR)	12.02 (2.95, 28.63)	17.98 (9.06, 79.58)	0.053
PICU LOS for those discharged alive, median days (IQR)	10.98 (2.70, 27.77)	14.39 (8.53, 43.98)	0.21
Overall hospital length of stay, median days (IQR)	22.23 (7.03, 38.6)	30.33 (13.89, 103.74)	**0.018**
Hospital LOS for those discharged alive, median days (IQR)	22.23 (6.48, 38.60)	27.96 (13.18, 89.64)	0.060

There was no significant difference in PICU LOS between the two groups (*p* = 0.053) or for the patients who were discharged alive (*p* = 0.21). However, hospital LOS was significantly longer in the long-course group (*p* = 0.018) (Table 4). In a multivariable regression analysis using mechanical ventilation and pulmonary disease as covariates this remained true (*p* = 0.037).

There was no statistical difference in previous MDROs between the groups (*p* = 0.50) or relationship between previous MDRO and new MDRO (*p* = 0.28). Ten percent of patients developed a new MDRO in our study with three patients in the short course and 6 in the longer course group. However, this difference was not statistically significant (*p* = 0.48). These MDROs included methicillin-resistant *Staphylococcus aureus,* multidrug-resistant *Pseudomonas aeruginosa, Stenotrophomonas maltophilia, Escherichia coli,* and *Enterobacter cloacae.*

## Discussion

Culture-negative sepsis remains an entity to be elucidated. The majority of adult literature focuses on characteristic and outcome comparisons of culture-negative and culture-positive sepsis with conflicting results. Gupta et al found that patients with culture-negative sepsis were more likely to have co-morbidities, acute organ dysfunction, and higher mortality.^8^ Phua et al. found patients with culture-negative sepsis to have fewer co-morbidities and shorter duration of hospitalization.^9^ Yet, additional studies suggest similar characteristics and outcomes between culture-positive and culture-negative sepsis events.^10,11^

When it comes to treatment of culture-negative sepsis, data are even more limited, especially in the pediatric population. Our results suggest that treatment of culture-negative sepsis for less than or equal to 7 days may be adequate for patients in the pediatric ICU based on the outcomes of mortality and length of stay.

Interestingly, patients who have been hospitalized for a longer period before the sepsis event, those on mechanical ventilation, and underlying pulmonary disease were more likely to be treated with a longer course of antibiotics. However, mechanical ventilation and underlying pulmonary disease were not shown to be confounding variables, however, in our primary outcome of hospital length of stay suggesting that length of antimicrobial treatment could be a contributing factor in prolonging hospitalization.

We hypothesize that there is a perception that patients with longer prior hospitalization, requiring mechanical ventilation, or underlying pulmonary disease are more sick, not improving, or higher risk for a serious bacterial infection persuading providers to extend the course of antibiotics.

Despite similar severity of illness scores between the two groups, it is possible that these patients had more severe illness at the time of the sepsis event. This may be supported by the increased overall mortality rate in the longer course group. The PIM-2 and PRISM 3 scores are reflections of the patients at admission. Given the longer course group was more likely to be hospitalized for a longer period of time prior to the sepsis event, these mortality risk scores may have changed since admission.

If the perception of severity of illness was higher in the long-course treatment group, one might expect the selection of antibiotics to change. However, in our study, the choice of antibiotics was similar between the two groups. Upon further analysis, there was a difference in DOTs between the two groups (Supplemental Figure 1). This may suggest that providers were hesitant to narrow antibiotics based on the perceived severity of illness, despite no identified pathogen or specific focus of infection. The assessment of unnecessary antimicrobials has been identified as a knowledge gap in the surviving sepsis campaign.^2^

One of the largest pediatric studies assessing the prevalence and outcomes of patients with severe sepsis found only 65% of patients had an identified organism on culture.^15^ In culture-negative sepsis, patients often remain on broad-spectrum antibiotics for prolonged periods of time because there is no organism for which to tailor therapy. Since unnecessary or prolonged antibiotic exposures contribute to the continued threat of MDROs, we assessed MDRO acquisition for our secondary outcome. Although our study sample was insufficiently powered for MDRO acquisition, notably, there were double the cases of new MDROs in the group treated with a long course of antibiotics. Like us, Tamma et al also observed an increase in MDRO acquisition among patients receiving longer durations of therapy.^14^ Furthermore, Tashome et al reported that additional antibiotic exposure can contribute to the development of resistance, with each additional day of exposure conferring a 4%–8% risk.^16^ Given the clinical risks of MDRO development, we believe identifying the shortest, effective duration of therapy for the treatment of culture-negative sepsis remains an important area of study with a larger population. Based on our current data, we calculated a sample size of 298 subjects needed to achieve 80% power.

As a single-center, retrospective study, there were inherent limitations. Identification of patients with culture-negative sepsis was difficult, but we did follow a validated data collection method for the identification of sepsis in hospitalized patients.^17^ ICD 9/10 codes are not a reliable source for routinely identifying culture-negative patients, and the rationale for antibiotic courses was not always clearly documented. Unlike other studies, we did exclude patients documented to have aspiration pneumonia with X-ray findings, pneumonia, cellulitis/skin/soft tissue infections, toxic shock syndrome, and endocarditis, despite negative cultures given current literature to aid in treatment duration for these conditions. Although this strengthens our study by focusing on patients with concerns for infection despite no obvious source, it does limit our sample size. One major limitation in our study is the variation in length of time patients were hospitalized prior to sepsis case, making it challenging to interpret hospital length of stay exclusively from sepsis event between the two groups. In addition, as a retrospective study, not every patient in our study had follow-up cultures further limiting our evaluation for subsequent MDRO acquisition. Also, we did not evaluate differences in treatment duration related to providers. Though there may be a provider effect, our large critical care medical staff (including 24 physicians, 18 advance practice providers, and 12 fellows) likely balances out any effect from one single provider. Furthermore, we were not able to include data related to mechanical ventilation or underlying pulmonary disease, both of which could contribute risk for infection, due to limitations with our data set. However, it is not standard practice at our institution to start antibiotics solely based on chest X-ray or ventilator changes and was more likely related to a constellation of symptoms, strengthening the real-world application of our findings.

In conclusion, there are limited data to guide the treatment of culture-negative sepsis, and thus, it is identified as a knowledge gap within the pediatric surviving sepsis campaign.^2^ Our single-center, retrospective study suggests patients treated with shorter courses of antibiotics do not have worse outcomes to those treated with longer courses and may have the benefit of reducing future development of resistant organisms. Future prospective studies with a larger population are warranted.

## Supporting information

Wehrenberg et al. supplementary material Wehrenberg et al. supplementary material

## Supplementary material

For supplementary material accompanying this paper visit http://doi.org/10.1017/ash.2023.502. click here to view supplementary material

## Research transparency and reproducibility

N/A.
