# Supplementary material for: Antibiotic treatment duration for culture-negative sepsis in the pediatric intensive care unit
Source: Antimicrob Steward Healthc Epidemiol. 2023 Dec 22;3(1):e249. doi: 10.1017/ash.2023.502 (PMC10753480; doi:10.1017/ash.2023.502)
Supplement: Wehrenberg et al. supplementary material [file S2732494X23005028sup001.pdf]

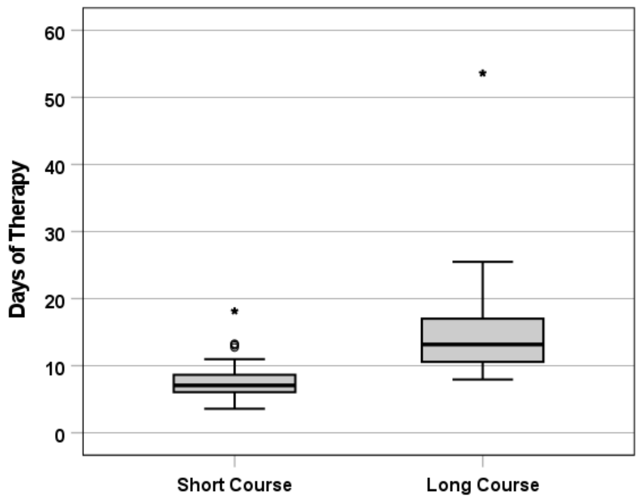

Supplemental figure 1: Comparison of days of therapy (DOT) between short (less than or equal to 7 days) and long (greater than 7 days) courses of antibiotics
